# Biomechanical Analysis of the Efficacy of Locking Plates during Cyclic Loading in Metacarpal Fractures

**DOI:** 10.1155/2014/648787

**Published:** 2014-03-13

**Authors:** Stefanie Doht, Rainer H. Meffert, Michael J. Raschke, Torsten Blunk, Sabine Ochman

**Affiliations:** ^1^Department of Trauma, Hand, Plastic and Reconstructive Surgery, University Clinics of Wuerzburg, Oberduerrbacher Straße 6, 97080 Wuerzburg, Germany; ^2^Department of Trauma, Hand and Reconstructive Surgery, University Clinics of Muenster, Waldeyerstraße 1, 48149 Muenster, Germany

## Abstract

*Purpose.* To analyse the biomechanical characteristics of locking plates under cyclic loading compared to a nonlocking plate in a diaphyseal metacarpal fracture. *Methods.* Oblique diaphyseal shaft fractures in porcine metacarpal bones were created in a biomechanical fracture model. An anatomical reduction and stabilization with a nonlocking and a comparable locking plate in mono- or bicortical screw fixation followed. Under cyclic loading, the displacement, and in subsequent load-to-failure tests, the maximum load and stiffness were measured. *Results.* For the monocortical screw fixation of the locking plate, a similar displacement, maximum load, and stiffness could be demonstrated compared to the bicortical screw fixation of the nonlocking plate. *Conclusions.* Locking plates in monocortical configuration may function as a useful alternative to the currently common treatment with bicortical fixations. Thereby, irritation of the flexor tendons would be avoided without compromising the stability, thus enabling the necessary early functional rehabilitation.

## 1. Introduction

Metacarpal and phalangeal fractures are the most common fractures of the upper extremity [1]. Around 50% of the hand fractures appear in young adults (15–34-year-old) [2]. The architecture of the metacarpus is crucial for the fine mechanics of the hand and fingers [3]. Thus, an anatomical reduction of metacarpal fractures is needed to provide a good clinical outcome and to restore hand function. Undisplaced metacarpal fractures may be treated conservatively with cast immobilization in intrinsic plus position for four to six weeks. Dislocated fractures and fractures with malrotation have to be treated operatively [4, 5]. Intramedullary k-wires, screws, or a dorsal plate osteosynthesis are the common options for stabilization. However, especially plate fixation is associated with several problems. On the one hand, the flexor tendon sheaths directly below the bones can be easily irritated by bicortical drilling and screwing, which is the standard method for plate fixation in hand surgery. On the other hand, the close contact of the plate to the extensor tendons bears a high risk of adhesions, which, postoperatively and after immobilization, may be the reason for a bad outcome and limited hand function. Thus, early controlled active and passive motion of the fingers are requested after an operative treatment [6, 7]. A high stability has to be maintained in order to allow a short time of immobilization and an early functional treatment.

Comparing the different techniques of osteosynthesis for metacarpal fracture stabilization the combination of a dorsal plate and lag screw has been shown in biomechanical studies to be stronger than other fixation methods [8]. For a few years, locking plates have been used in hand surgery as well as in trauma surgery of the long bones. Recent biomechanical studies investigated the stability in load-to-failure tests [9–11]. However, regarding the importance of an early controlled passive motion in metacarpal fractures, additional biomechanical analysis of the stability of locking plates under cyclic loading is needed.

Therefore, the aim of this study was to determine the biomechanical properties of locking plates with a mechanism of interlocking by drilling titan in titan with different grades (Smart Lock) [12] compared to a nonlocking plate. More specifically, we analyzed both plates in bicortical as well as monocortical screw fixation. We hypothesized that with the locking plates even a monocortical screw fixation provides sufficient stability, which clinically would avoid the need for bicortical fixation with its associated irritation of the flexor tendons during treatment.

## 2. Materials and Methods

### 2.1. Specimen

Fresh frozen second metacarpal bones from domestic pigs (mean age 10 months) were used. The biomechanical properties of these bones correspond to human metacarpal bones and proximal phalanges with minimal interspecies variations of structure compared to human cadaver [13]. Using pig metacarpal bones has been validated in several studies to test the biomechanical features of hand fixation [9, 10, 13]. The specimens were dissected from soft tissue. Their physical properties (diameter, length, and cortical thickness) were measured to confirm that the test samples were similar and after wrapping within normal saline-soaked gauze stored at −70°C to preserve their biomechanical properties close to those of fresh bones. Before use, specimens were defrosted at room temperature of 20°C and embedded in a fixation device (14 mm) using Palacos (Kulzer GmbH, Wehrheim, Germany). All specimens were kept moist with saline irrigation at room temperature during preparation, the surgical procedure, and biomechanical testing to prevent desiccation.

### 2.2. Fracture Generation

Under physiological conditions, metacarpal bones underlie bending forces. Thus, we simulated the physical bending stress by a modified three-point bending test setup and generated an oblique midshaft fracture in a biomechanical fracture model using an electromechanical uniaxial testing machine (Zwick/Roell, Z005/TN2A, Ulm, Germany) as described previously [14]. Maximum load and stiffness of the native bones were measured.

### 2.3. Biomechanical Test Setup

Biomechanical testing was performed using the same three-point bending test setup as mentioned above. In order to determine the experimental conditions, a pretesting series was performed. The simulated metacarpal fractures were reduced and stabilized with a nonlocking plate in a bicortical screw fixation. For the pretests, specimens were randomly divided into five groups with five specimens per group. Knowing the maximum load of the native bone, cyclic loading with 1000 cycles was performed with 10% of the maximum load of each native bone in group 1, 20% in group 2, 30% in group 3, 40% in group 4, and 50% in group 5. The mean displacement in group 1 was 0.64 ± 0.27 mm and in group 2 0.91 ± 0.57 mm. In group 3, two of the five bones failed before completing the 1000 cycles. The mean displacement of group 3 was 1.58 ± 0.35 mm. In group 4, four of five and in group 5 every specimen failed before completing the 1000 cycles.

According to these findings, for the actual experiment, cyclic loading was performed with 20% of the maximum load of each native bone. For testing, the specimens were first loaded with 1 to 10 N for ten settling cycles. Then, 1000 cycles with 1 Hz were applied from 10 N to 20% of the maximum load of the native bone. In the two most promising groups, II and III (see below), after cyclic loading additionally a load-to-failure test was performed. Data collection during mechanical testing was done using testXpert V10.11.

### 2.4. Experimental Groups

For all specimens, after reduction, the oblique shaft fractures were stabilized with a dorsal plate osteosynthesis. Two different plates (thickness 1 mm) were used: a 4-hole nonlocking plate with a linear configuration and 2.3 mm screws (Leibinger-Stryker, Freiburg, Germany); a comparable 4-hole locking plate with also a linear configuration and 2.0 mm screws (Variax Stryker, Freiburg, Germany). With these locking plates, interlocking is achieved using the Smart Lock technology: Grade V titanium screws/pegs (harder) are drilled into the circular lips (Grade II titanium (softer) of the plate holes [12]. Forty specimens were divided into four groups with ten metacarpals per group. Group size was calculated by a power analysis. After reduction, the metacarpal fractures were stabilized with a nonlocking plate with four monocortical screws (group I), a nonlocking plate with four bicortical screws (group II), a locking plate with four monocortical screws (group III), and a locking plate with four bicortical screws (group IV). All fixations were done by the same experienced surgeon according to a standardized technique.

### 2.5. Statistical Analysis

Maximum load, stiffness, and displacement were compared with the nonparametric Mann-Whitney *U* test using SPSS. Statistical significance was set at a value of  *P* < 0.05.

## 3. Results

### 3.1. Cyclic Loading

The mean load which was applied during cyclic loading was similar in all groups (20% of the maximum load of each native bone). The mean load of all specimens was 110.21 ± 29.42 N, the mean load of each group is shown in Table 1. In group Ione of the ten specimens failed before completing the 1000 cycles. In the other groups all fracture constructs survived all phases of the cyclic loading testing.

**Figure 1:**
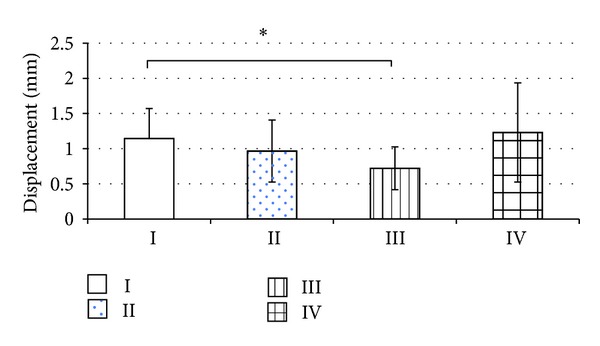
The displacement under cyclic loading (1,000 cycles) was determined for all experimental groups, that is, the locking and nonlocking plates, either in monocortical or bicortical screw fixation. ∗ denotes statistical significance, *P* < 0.05.

With regard to the displacement, a significant difference between the monocortical screw fixation groups was demonstrated, with a lower displacement achieved with the locking plate (group I > group III, *P* = 0.028) (Figure 1). For the monocortical locking plate (group III), also a trend of a lower displacement was observed compared to bicortical fixation with either nonlocking (group II, *P* = 0.315) or locking plate (group IV, *P* = 0.075); however no significance was reached (Figure 1). Similar results were obtained with mono- and bicortical fixation using the nonlocking plate (groups I and II, *P* = 0.315) and with bicortical fixation using either the nonlocking or the locking plate (groups II and IV, *P* = 0.529) (Figure 1).

During the cyclic loading, the specimens were examined macroscopically. The displacement was an invisible bending and did not lead to a loosening of locking mechanism or the implant-bone interface.

### 3.2. Load-to-Failure Tests

**Figure 2:**
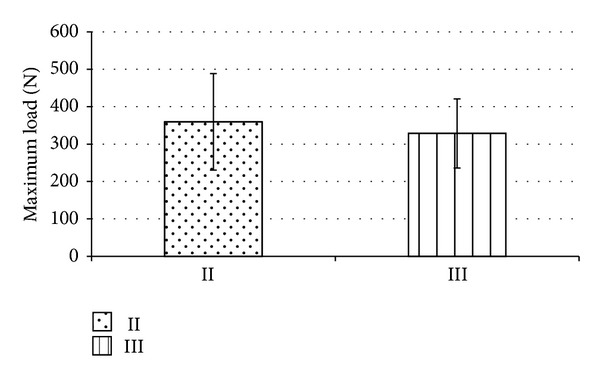
The maximum load (load-to-failure after cyclic loading) was determined for group II (bicortical nonlocking plate) and group III (monocortical locking plate). No statistically significant difference was found.

**Figure 3:**
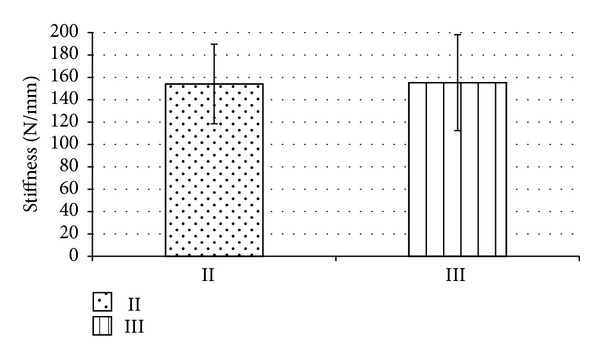
The stiffness was determined for group II (bicortical nonlocking plate) and group III (monocortical locking plate). No statistically significant difference was found.

Bicortical nonlocking (group II) and monocortical locking plate fixation (group III) were further compared regarding maximum load and stiffness after cyclic loading. For the maximum load, no significant difference could be demonstrated between group II (359.5 ± 129 N) and group III (328.6 ± 92.4 N) (Figure 2). Stiffness revealed similar results, that is, 154 ± 36 N/mm for bicortical nonlocking and 155 ± 43 N/mm for monocortical locking plate fixation (Figure 3).

The mode of failure of the nonlocking plate was a bending of the plate with a displacement at the fracture. The failure of the locking plate was at the screw-bone interface. The screws exhibited a pull-out at the bone. The screw-plate interface was stable and the locking mechanism was kept intact after the load-to-failure tests, also without plate deformation. The mode of failure was highly reproducible with almost the same type of failure in all specimens within each group.

## 4. Discussion

Metacarpal fractures are a common injury of the upper extremity and usually occur in young adults. The metacarpus is essential for the anatomical structure of the hand and for the motion of the fingers. Malposition and malrotation have a large negative effect on the fine mechanics of the hand. Thus, an operative treatment has to stabilize the fracture fragments in an anatomical position. By the close contact of the extensor tendons and the dorsally fixed plate osteosynthesis, postoperative adhesions may appear. To minimize the risk of postoperative limitation of the range of motion, an early functional treatment is required [6, 7]. Feehan et al. demonstrated advantages of early controlled passive motion to reduce fracture dorsal angulation and to increase the stability during fracture healing [7].

To allow an early functional treatment, a high primary stability has to be maintained. Comparing the different options for fracture stabilization, a dorsally fixed plate osteosynthesis in combination with an interfragmentary screw was shown to provide the highest stability [8, 15, 16]. Prevel et al. demonstrated a higher stability for 2.3 mm screws compared to 1.2 and 1.7 mm screws in linear 4-hole plate fixation [17]. More recently, the biomechanical properties of bioabsorbable plates have been determined. They provide a comparable stability to the commonly used nonabsorbable plates [18]. However, the absorbable implants did not lead to a new standard in hand traumatology due to the risk of foreign-body reaction [19].

As in fracture fixation of the long bones, locking plates were also available for hand surgery in many variations. Recent biomechanical studies analysed the stability of these plates in load-to-failure tests. Gajendran et al. demonstrated a higher stability for double-row locking plates compared to nonlocking plates in comminuted metacarpal fractures [11]. An equal stability for double-row locking plates only in a monocortical screw fixation compared to bicortical nonlocking linear plates could be shown for the polyaxial angular stable TriLock system [10]. Plates with the interlocking mechanism of titan deformation, which are also used in this study, provided a higher stability with locking monocortical screws compared to nonlocking plates [9]. All of these previous studies have been restricted to load-to-failure analysis. However, taking into account the high importance of an early functional treatment, there is a strong need for biomechanical studies to determine the different stabilities of locking and nonlocking plates under cyclic loading.

A recent biomechanical study analysed nonlocking plates with mono- and bicortical screws in a three-point bending test under cyclic loading in osteotomized metacarpals [20]. The loads were applied with 100 N for 10 cycles and after that raised for 100 N every 10 cycles. Already at 200 N, the yield load was reached and some metacarpals failed. For nonlocking plates, the bicortical screw fixation was shown to be advantageous [20].

In contrast to this report, in our study, using only 20% of the maximum load of native bone, continuous cyclic loading for 1000 cycles was possible with observation of differential displacement but without immediate failure.

Under cyclic loading, that is, simulating the repetitive bending forces during early functional finger movements, similar displacement was observed for mono- or bicortical screw fixation, using either nonlocking or locking plates. In fact, there was a trend for the locking plates in monocortical fixation to yield the lowest displacement in this study. In load-to-failure tests after cyclic loading, locking plates in monocortical screw fixation and nonlocking plates in bicortical screw fixation yielded very similar results. These findings are well in agreement with an earlier study of our group investigating the same implants with regard to load-to-failure only, that is, without previous cyclic loading [9]. Also under those conditions, no differences could be detected between locking plates in monocortical configuration and nonlocking plates in bicortical configuration [9].

## 5. Conclusions

The results of our biomechanical study may have an immediate clinical relevance suggesting that with the use of a locking plate a monocortical screw fixation can be an alternative to nonlocking plates with bicortical screws. This configuration avoids irritation of the flexor tendons without compromising the stability, thus enabling the necessary early functional rehabilitation.

## Figures and Tables

**Table 1:** Mean load (*F*) applied during cyclic testing and displacement measured for the locking and nonlocking plates.

	Mean	SD
Nonlocking plates		
Monocortical (group I) (*n* = 10)		
*F* [N]	101.6	28.4
Displacement [mm]	*1.14 *	*0.45 *
Bicortical (group II) (*n* = 10)		
*F* [N]	119	28.67
Displacement [mm]	*0.96 *	*0.44 *

Locking plates		
Monocortical (group III) (*n* = 10)		
*F* [N]	112.8	17.96
Displacement [mm]	*0.72 *	*0.30 *
Bicortical (group IV) (*n* = 10)		
*F* [N]	107.4	40.19
Displacement [mm]	*1.23 *	*0.70 *
